# The VP3 Factor from Viruses of *Birnaviridae* Family Suppresses RNA Silencing by Binding Both Long and Small RNA Duplexes

**DOI:** 10.1371/journal.pone.0045957

**Published:** 2012-09-25

**Authors:** Adrian Valli, Idoia Busnadiego, Varvara Maliogka, Diego Ferrero, José R. Castón, José Francisco Rodríguez, Juan Antonio García

**Affiliations:** Centro Nacional de Biotecnología-CSIC, Madrid, Spain; National University of Singapore, Singapore

## Abstract

RNA silencing is directly involved in antiviral defense in a wide variety of eukaryotic organisms, including plants, fungi, invertebrates, and presumably vertebrate animals. The study of RNA silencing-mediated antiviral defences in vertebrates is hampered by the overlap with other antiviral mechanisms; thus, heterologous systems are often used to study the interplay between RNA silencing and vertebrate-infecting viruses. In this report we show that the VP3 protein of the avian birnavirus *Infectious bursal disease virus* (IBDV) displays, in addition to its capacity to bind long double-stranded RNA, the ability to interact with double-stranded small RNA molecules. We also demonstrate that IBDV VP3 prevents the silencing mediated degradation of a reporter mRNA, and that this silencing suppression activity depends on its RNA binding ability. Furthermore, we find that the anti-silencing activity of IBDV VP3 is shared with the homologous proteins expressed by both insect- and fish-infecting birnaviruses. Finally, we show that IBDV VP3 can functionally replace the well-characterized HCPro silencing suppressor of *Plum pox virus*, a potyvirus that is unable to infect plants in the absence of an active silencing suppressor. Altogether, our results support the idea that VP3 protects the viral genome from host sentinels, including those of the RNA silencing machinery.

## Introduction

RNA silencing collectively refers to diverse gene expression regulatory pathways that control a large number of cellular processes, such as developmental patterning, responses to biotic and abiotic stresses, and maintenance of genome stability. It is conserved in animals, plants, most fungi and some protists [1]–[3]. RNA silencing pathways involve small RNAs of 20–30 nucleotides, which are mainly derived from digestion of RNA duplexes by the action of RNase III–like nucleases called Dicer or Dicer-like (DCL) proteins [4]–[6]. These small RNAs are then associated with effector complexes, containing a protein belonging to the Argonaute (AGO) family, to guide sequence specific mRNA degradation, translational inhibition, or epigenetic modifications [7]. Interestingly, it has been well-established that RNA silencing plays a key antiviral role in plants, fungi and invertebrate animals, where infecting viruses induce the production of viral-derived small interfering (si)RNAs from their genomes, replication-intermediate double-stranded (ds)RNAs, fold-back structures within viral mRNAs, or dsRNA molecules produced by the action of RNA-dependent RNA polymerases (RdRps) on a single stranded viral RNA template [8]–[11]. Although the role of RNA silencing as an antiviral mechanism in vertebrate animals is controversial, the fact that *bona fide* viral-derived small RNAs from different mammal-infecting viruses have recently been identified suggests that RNA silencing could also be an antiviral strategy in these organisms [12]–[14].

Viruses have evolved to overcome RNA silencing-based defences by encoding viral suppressors of RNA silencing (VSR) [15]–[18]. The key relevance of these proteins for the infection life cycle has been established in multiple virus/host combinations, showing that the absence of an active VSR results in greatly reduced viral infection [19], [20]–[25]. Interestingly, it has been found that several vertebrate-infecting viruses also encode proteins with the ability to counteract RNA silencing, such as NS1 from *Influenza A virus* [26]–[29], VP30, VP35 and VP40 from *Ebola virus* [28], [30], [31], NSs from *La Crosse virus* [32], σ3 from *Mammalian orthoreovirus type 3* [33], E3L from *Vaccinia virus* [28], [29], Tas from *Primate foamy virus type 1* [34], Tat from *Human immunodeficiency virus* [35], Rex from *Human T-cell lymphotropic virus* [36], C and E2 proteins from *Hepatitis C virus* [37]–[39], 7a from *Severe acute respiratory syndrome virus* [40], and HVT063 from *Turkey herpesvirus* [41], presumably as part of the attack-defense-counterdefense arms race between viruses and their hosts (some of these RSSs were recently reviewed [42], [43]–[46]).

*Birnaviridae* is a family of non-enveloped icosahedral viruses that infect birds, fishes, insects or rotifers [47]. Two members of the family, namely *Infectious bursal disease virus* (IBDV) and *Infectious pancreatic necrosis virus* (IPNV), are etiological agents of diseases imposing a heavy economical burden on the poultry and aquaculture industries, respectively. IBDV causes a highly acute immunosuppressive disease affecting juvenile domestic chickens [48]. IPNV is responsible for an acute systemic disease affecting different species of freshwater and marine fishes, mollusks, and crustaceans [49]. Birnaviruses contain a polyploid bipartite dsRNA genome that is packaged into a single virus particle [50]. Structural units are derived from a polyprotein precursor that is translationally self-cleaved to release three polypeptides, pVP2 (the capsid protein precursor), VP4 (the protease) and VP3 [51]. VP3 is a multitasking protein that has several activities throughout the viral life cycle. In addition to being a self-interacting protein [52], VP3 interacts with pVP2 during particle morphogenesis [53], with VP1 acting as a transcriptional activator [54], and with the dsRNA to make ribonucleoprotein complexes [55], a unique feature among dsRNA viruses. The atomic structure of VP3 was partially solved by X-ray crystallography to 2.3 Å [56].

The genome of most dsRNA viruses is contained within a specialized icosahedral capsid involved in transcription and replication of the dsRNA genome [57]. The structural integrity of these functional cores remains undisturbed after virus entry in the infected cell [58], protecting the dsRNA and replicative intermediates from host defense mechanisms. In contrast, birnaviruses lack this replicative core [59], [60], but riboncleoprotein complexes might have acquired some of its defensive functions.

Here, we report that different birnaviral VP3 proteins suppress the RNA silencing of a GFP reporter gene and that, despite their ability to bind ds-siRNAs, they appear to use a different mechanism than that of the well-studied short interfering (si)RNA-hijacking suppressors. In addition, we show that IBDV VP3 is able to functionally replace the well-characterized HCPro VSR in the plant-infecting *Plum pox virus* (PPV), which highlights the silencing suppression activity of VP3 in the context of a highly sensitive infection process.

## Results

### IBDV VP3 Suppresses Both Sense RNA and dsRNA-triggered RNA Silencing in Plants

**Figure 1:**
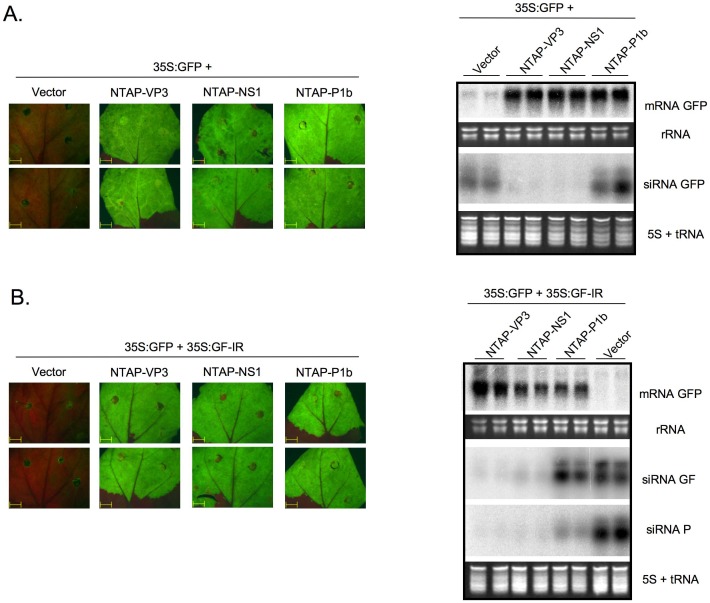
VP3 protein from IBDV suppresses the RNA silencing. ssRNA-triggered (A) and dsRNA-triggered (B) silencing assays in *N. benthamiana* plants. The figure shows GFP fluorescence pictures taken under an epifluorescence microscope using the same exposure time (left panels), and Northern blot analyses of GFP mRNA and GFP-derived siRNAs (right panels) in agroinfiltrated leaf patches from two plants expressing the indicated constructs, and collected at 6 days post agroinfiltration. Two different probes were used for detection of GFP siRNAs: the GF probe (for primary plus secondary siRNAs) corresponds to the GFP fragment included in the inverted repeat RNA expressed from the silencing trigger plasmid p35S:GF-IR; and the P probe (specific for secondary siRNAs) corresponds to the 3′ terminal region of the GFP gene, which is not included in p35S:GF-IR. EtBr-stained rRNA and 5S+tRNA are shown as loading controls.

The high affinity displayed by IBDV VP3 to bind long dsRNA molecules as well as to protect them from RNase III-mediated cleavage *in vitro* [55], prompted us to test whether IBDV VP3 could suppress RNA silencing *in vivo*. For this, we decided to use an heterologous transient expression system in *Nicotiana benthamiana* [15], as it provides readout of RNA silencing without the confounding induction of other immune and interferon-responsive pathways [61], [62]. Different plasmids expressing untagged and N-terminal TAP (NTAP)-tagged versions of IBDV VP3 were constructed and transferred to *Agrobacterium tumefaciens*. Equivalent constructs expressing Influenza A virus NS1 and the well-characterized CVYV P1b silencing suppressor [63], [64] were used as positive controls. A GFP-expressing plasmid (p35S:GFP) was used as the inducer, target and reporter of RNA silencing. For simplicity, we will refer to *Agrobacterium* strains by the name of the plasmid they carry. Leaf patches infiltrated with p35S:GFP plus either pMDC32 or pNTAPi empty vectors displayed the highest intensity of GFP fluorescence at 2–3 days post-agroinfiltration (dpa) (not shown). GFP fluorescence in these leaves dropped to hardly detectable levels by 6 dpa (Fig. 1A and not shown) and, consistently with this fact, Northern blot analysis showed very low accumulation of GFP mRNA in them (Fig. 1A and data not shown). In contrast, leaves infiltrated with p35S:GFP plus pMDC32-NS1, p35S-NTAP-NS1 or p35S-NTAP-P1b, expressing untagged or TAP-tagged NS1, or TAP-tagged P1b, respectively, showed bright fluorescence at 6 dpa given the protection of GFP mRNAs from degradation by post-transcriptional gene silencing (Fig. 1A and data not shown). Interestingly, those leaf patches infiltrated with p35S:GFP plus either pMDC32-VP3 or p35S-NTAP-NS1 expressing untagged or TAP-tagged VP3, respectively, displayed GFP fluorescence as bright as positive controls at 6 dpa. Northern blot analysis revealed that the expression of IBDV VP3 inhibited GFP mRNA degradation very efficiently (Fig. 1A and data not shown).

To induce RNA silencing, the GFP mRNA is firstly converted to dsRNA. In order to better understand the mechanism by which IBDV VP3 suppresses RNA silencing, a third *Agrobacterium* strain expressing an inverted repeat (IR) that directly generate dsRNA from the 5′ region of the GFP gene (p35S:GF-IR) was added to the infiltrated mixes. Given that GF-IR directed a fast and strong silencing of the GFP mRNA reporter, no fluorescence was detected in the infiltrated patches at 6 dpa in the absence of silencing suppressors, and consistent with this observation, a Northern blot assay showed no accumulation of GFP mRNA (Fig. 1B). In contrast, those patches expressing NTAP-VP3, NTAP-NS1 or NTAP-P1b displayed strong fluorescence and high accumulation of GFP mRNA as consequence of their ability to protect the reporter mRNA from silencing also in this assay (Fig. 1B).

### IBDV VP3 and Influenza Virus NS1 Strongly Reduce the Generation of Primary siRNAs

Accumulation of specific siRNAs is a main hallmark of RNA silencing induction, and it can be observed in the Northern blot analyses of leaf patches infiltrated with either p35S:GFP or p35S:GFP plus p35S:GF-IR, together with the empty vector (Fig. 1A and B). In the sense RNA-triggered silencing assay, NTAP-P1b had, as previously reported [63], very little effect on GFP siRNA accumulation (Fig. 1A). In contrast, both NTAP-NS1 and NTAP-VP3 proteins appeared to abolish the generation of GFP siRNAs (Fig. 1A). Similarly, in the dsRNA-triggered silencing assay, NTAP-P1b had no effect on the generation of siRNAs from the 5′ terminal two thirds of the GFP gene (siRNA GF), which derive mainly from the dsRNA trigger (p35S:GF-IR). However, the accumulation of siRNAs from the 3′ terminal region of the GFP sequence, which are not encoded by p35S:GF-IR and, thus, are secondarily generated in the amplification phase of the RNA silencing process (siRNA P), is strongly inhibited by NTAP-P1b (Fig. 1B and [64]). Interestingly, expression of either NTAP-VP3 or NTAP-NS1 produced a strong reduction in the generation not only of P-derived siRNAs, but also of those siRNAs deriving from the GF dsRNA region (Fig. 1B), indicating that these proteins use a mechanism different from that of CVYV P1b to suppress the RNA silencing.

### VP3 Efficiently Binds ds-small RNAs

**Figure 2:**
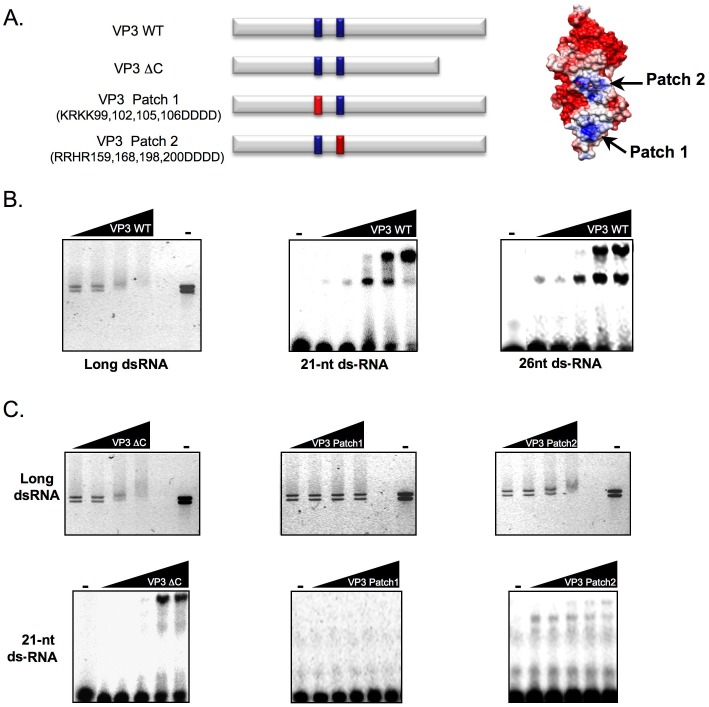
IBDV VP3 binds long and short dsRNAs, and the positively charged domain Patch 1 is involved in this interaction. (A) Schematic representation of wild type IBDV VP3 and derivative mutants used in this assay (red bars indicate mutated patches). The distribution of electrostatic potential on VP3 surface (adapted from [56]) is shown at the right. Both Patch 1 and Patch 2 positively charged regions are indicated in blue, whereas negatively charged regions of the protein are shown in red. (B) (Left panel) Purified IBDV VP3 WT protein (final concentration of 10, 20, 40 and 80 nM) was incubated with the IBDV genomic dsRNAs. (Central and right panels) Purified IBDV VP3 protein (final concentration of 80, 160, 320, 640 and 1200 nM) was incubated with ^32^P-labelled 21-nt or 26-nt ds small RNAs. (C) (Upper panels) Purified VP3 ΔC (final concentration of 12, 24, 48 and 96 nM) and Patch 1 or Patch 2 mutant VP3 (final concentration of 10, 20, 40 and 80 nM) were incubated with IBDV genomic dsRNAs. (Lower panels) Purified VP3 ΔC (final concentration of 90, 180, 360, 720 and 1440 nM), and Patch 1 or Patch 2 mutant VP3 (final concentration of 80, 160, 320, 640 and 1200 nM) were incubated with ^32^P-labelled 21-nt small RNAs. In both B and C, protein-IBDV genomic dsRNA complexes were resolved in agarose gels, stained with EtBr, and photographed under UV light, and protein-small RNA complexes were resolved in polyacrylamide gels and revealed by autoradiography.

It was previously reported that birnaviral VP3 proteins, including IBDV VP3, are able to bind dsRNAs molecules [65]–[67]. In order to test the ability of IBDV VP3 to interact with typical siRNA duplexes, EMSAs using ^32^P-labelled synthetic ds-siRNAs carrying 2-nt overhand at their 3′ ends were carried out. For these experiments, a 6xHis-tagged version of IBDV VP3 was produced and purified from insect cell cultures making use of recombinant baculoviruses. Purified VP3 samples were incubated with different dsRNA molecules, and complexes were then resolved by gel electrophoresis (Fig. 2B). As expected from previous reports, purified IBDV VP3 was able to bind IBDV genomic dsRNA molecules producing a typical smear-like gel shifting of the dsRNA probe akin to that observed in isolated IBDV ribonucleoprotein complexes (Fig. 2B and [55]). Importantly, this protein was also able to interact with siRNA duplexes of both 21- and 26-nt of length, without apparent size specificity (Fig. 2B).

### dsRNA Binding is Crucial for VP3-mediated RNA Silencing Suppression

The relevance of interactions between VSRs and diverse RNA molecules in silencing pathways has been previously reported (reviewed in [15], [18]). A mutagenic approach was followed to assess the importance of VP3-dsRNA interactions in the VP3 silencing suppression activity. Positively charged amino acids of the IBDV VP3 protein, predicted from crystal structure [56] to be from the region involved in dsRNA binding, were replaced by negatively charged Asp residues (Patch 1 and Patch 2, Fig. 2A). In addition, a deletion of the highly hydrophilic C-terminal region of IBDV VP3 (position 222–258), which was previously proposed as potentially relevant in both nucleic acid and protein-protein interactions [56], was engineered (ΔC, Fig. 2A). Mutant proteins were produced and purified from insect cells, and tested for their dsRNA binding capacity against the IBDV genomic RNAs, as well as a ^32^P-labelled 21-nt siRNA duplex, by EMSA (Fig. 2C).

None of the multiple substitutions of positively charged amino acids affected the capacity of IBDV VP3 to form dimers (Fig. S1), suggesting that these mutations do not cause a drastic disturbance of the global structure of the protein. The VP3 Patch 2 mutant caused a band shift of both long dsRNA and ds-siRNA, although with less efficiency than the wild type VP3 (Fig. 2C), suggesting that the patch 2 domain could have an ancillary contribution to the RNA binding. In contrast, both VP3 Patch 1 (Fig. 2C) and VP3 Patch 1+2 (data not shown) appear to be completely unable to reduce the mobility of long dsRNA and ds-siRNA, suggesting that VP3 Patch 1 plays a central role in binding to any species of dsRNA. The fact that VP3 ΔC causes shifts of long dsRNA and ds-siRNA in similar manner than those produced by the wild type protein (Fig. 2C) suggests that the C-terminal part of VP3 is not relevant for RNA binding under the experimental conditions used here.

**Figure 3:**
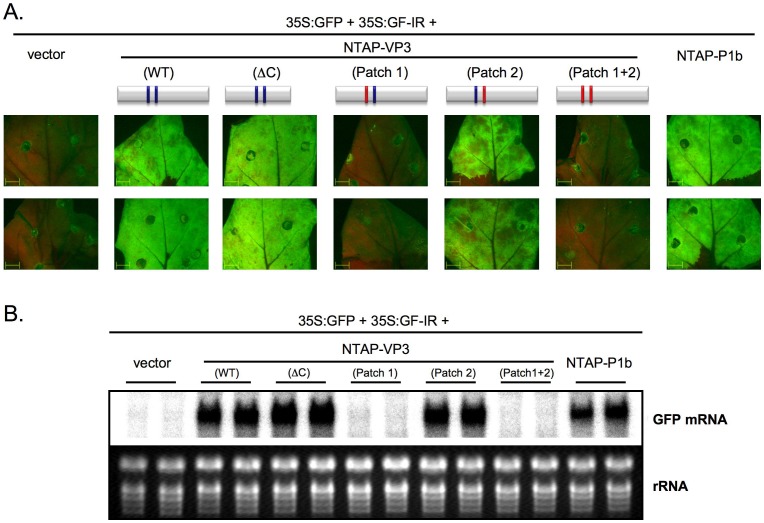
Positive correlation between dsRNA binding and RNA silencing suppression activity revealed by a dsRNA-triggered silencing suppression test. GFP fluorescence pictures taken under an epifluorescence microscope (A) and Northern blot analysis of GFP mRNA accumulation (B) of leaves of two *N. benthamiana* plants infiltrated with agrobacteria carrying the plasmid indicated above each lane (red bars indicate mutated patches). EtBr-stained rRNA is shown as loading control.

Patch 1, Patch 2 and ΔC mutations were engineered in pNTAP-VP3, and the effect of these modifications on the RNA silencing suppression activity of VP3 was assessed in a dsRNA-triggered silencing assay (Fig. 3). At 6 dpa, leaf patches expressing p35S:GFP, p35S:GF-IR, and either pNTAP-VP3, pNTAP-VP3Patch2, pNTAP-VP3ΔC or the positive control pNTAP-P1b displayed very high fluorescence levels (Fig. 3A). Northern blot analysis confirmed an efficient protection of GFP mRNA from silencing degradation by the VP3Patch2 and VP3ΔC mutants. In contrast, leaf patches expressing p35S:GFP, p35S:GF-IR, and either pNTAP-VP3Patch1 or pNTAP-VP3Patch1+2 showed no GFP fluorescence at 6 dpa, like control plants expressing p35S:GFP, p35S:GF-IR and an empty vector, suggesting that Patch1 mutation abolishes the VP3 anti-silencing activity. Northern blot analysis confirmed the fluorescence data since GFP mRNA was cleared from those tissues expressing VP3Patch1 or VP3Patch1+2 mutants at this time (Fig. 3B). Collectively, these results show a strong correlation between dsRNA interaction and RNA silencing suppression, supporting the idea that VP3 uses its dsRNA binding ability to suppress the RNA silencing.

### Homologous VP3 Proteins from Bird-, Fly- and Fish-infecting Viruses Share the Ability to Suppress RNA Silencing

**Figure 4:**
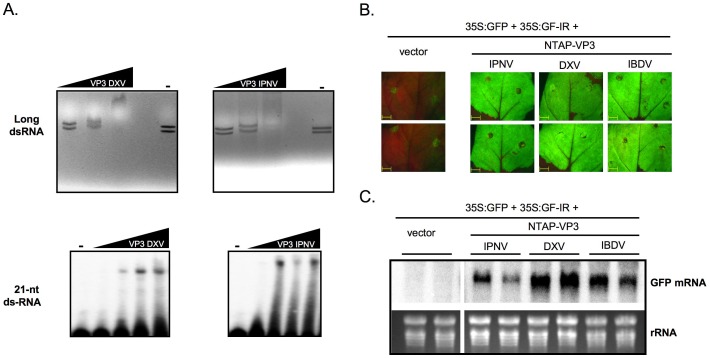
VP3 proteins from fish- and insect-infecting birnaviruses suppress RNA silencing in a dsRNA-triggered test. (A) (Upper panels) purified DXV VP3 (final concentration of 8.5, 17, 34 and 68 nM) and IPNV VP3 (final concentration of 10, 20, 40 and 80 nM) were incubated with IBDV genomic dsRNAs. Protein-dsRNA complexes were resolved in agarose gels, stained with EtBr, and photographed under UV light. (Lower panels) purified DXV VP3 (final concentration of 70, 140, 280, 560 and 1120 nM) and IPNV VP3 (final concentration of 80, 160, 320, 640 and 1280 nM) were incubated with ^32^P-labelled 21-nt ds small RNAs. Protein-small RNA complexes were resolved in polyacrylamide gels and revealed by autoradiography. (B) GFP fluorescence pictures taken under an epifluorescence microscope using the same exposure time, and (C) Northern blot analysis of GFP mRNA in agroinfiltrated leaf patches from two *N. benthamiana* plants expressing the indicated constructs, and collected at 6 days post agroinfiltration. EtBr-stained rRNA is shown as loading control.

The *Birnaviridae* family consists of viruses able to infect birds, such as IBDV, aquatic organisms and insects. To determine whether homologous proteins to IBDV VP3 from other birnaviruses infecting non-avian hosts are able to suppress RNA silencing, the VP3 coding sequences of IPNV and *Drosophila X virus* (DXV), which infect fishes and flies, respectively, were synthesized and introduced in the pFastBacHtb and NTAPi vectors to be expressed in insect cells and plants, respectively. IPNV and DXV VP3 proteins purified from insect cells were able to interact with the IBDV genomic dsRNAs, as well as a synthetic 21-nt ds-siRNA, by EMSA (Fig. 4A) in similar manner than their IBDV counterpart. The ability of these VP3 proteins to suppress RNA silencing was assessed in a dsRNA-triggered silencing assay in *N. benthamiana* plants. At 6 dpa, when leaf patches agroinfiltrated with p35S:GFP plus p35S:GF-IR and an empty vector showed strong silencing of GFP reporter, patches expressing p35S:GFP plus p35S:GF-IR and any of the NTAPi-derived plasmids expressing VP3 from either IBDV, IPNV or DXV displayed strong GFP fluorescence. Concomitantly, high accumulation levels of GFP mRNA were detected in agroinfiltrated leaf patches expressing these birnaviral proteins (Fig. 4B and C), indicating that both IPNV and DXV VP3s also suppress the RNA silencing.

### IBDV VP3 is able to Support PPV Infection in the Absence of its Natural Silencing Suppressor HCPro

**Figure 5:**
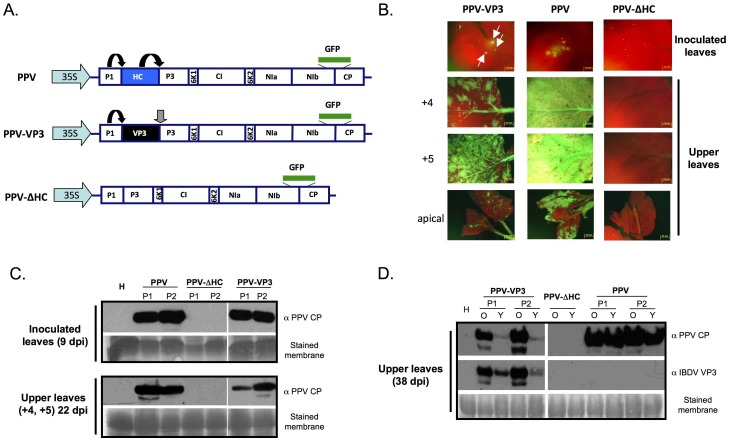
IBDV VP3 is able to functionally replace the HCPro silencing suppressor in a PPV infection. (A) Schematic representation of full-length cDNA clones derived from PPV and employed to infect *N. benthamiana* biolistically. The coding sequence of the GFP protein inserted between the NIb and CP cistrons is represented with a green rectangle. Black arrows indicate self-cleavages by the corresponding viral proteases, whereas the grey arrow indicates a cleavage *in trans* by the action of NIaPro. (B) GFP expression pattern of plants infected with the indicated viruses. Pictures of inoculated leaves collected at 7 days post inoculation (dpi), and the forth (+4) and fifth (+5) leaves above the inoculated one and the most apical leaves collected at 22 dpi were taken under an epifluorescence microscope. (C) Western blot analyses of plant tissue showing GFP foci collected at 9 dpi from inoculated leaves (upper panel) and at 22 dpi from upper non-inoculated leaves (+4 and +5 leaves) (lower panel) of two independent infected plants. (D) Western blot analysis of old (O) and young (Y) leaves collected at 38 dpi from two independent infected *N. benthamiana* plants. A polyclonal serum specific for PPV CP was used for assessment of virus accumulation, whereas immunoreaction with polyclonal sera specific for IBDV VP3 confirmed the identity of the infecting chimera. The membranes stained with Ponceau red showing the Rubisco are included as loading controls.

To assess the ability of IBDV VP3 to counteract an RNA silencing-based antiviral mechanism, we made use of a plant/virus pathosystem consisting of *N. benthamiana* as host and the potyvirus PPV as pathogen. A PPV full-length cDNA clone was engineered to obtain a chimera expressing IBDV VP3 instead of its natural VSR HCPro (PPV-VP3, Fig. 5A). HCPro is expressed as part of a genomic-length polyprotein, from which it is exscinded by the serine proteinase activity of the upstream protein P1 and its own cisteine proteinase activity [68]. To allow the excision of VP3, which lacks auto-proteolytic activity, from the viral polyprotein, both ends of the VP3 coding sequence cloned in the PPV chimera were slightly modified to generate cleavage sites recognized by P1 and NIa, the third PPV proteinase. Thus, the VP3 produced by PPV-VP3 is expected to have two extra aa (SD) at its N-end and a QVVVHQ tail at its C-end.

Plants were biolistically inoculated with cDNA of PPV-VP3, as well as wild type PPV and HCPro deletion mutant (PPV-ΔHC) clones, which were used as positive and negative control, respectively. The infection processes were monitored by visual inspection under visible light and under a fluorescence microscope, taking advantage of the GFP tag expressed by the three viruses. As expected, at 7 days post-inoculation (dpi), GFP foci were observed in all the leaves that had been inoculated with wild type PPV, whereas no foci appeared in leaves of plants inoculated with PPV-ΔHC. Interestingly, GFP foci also appeared at the same time in leaves of all plants inoculated with PPV-VP3 (Fig. 5B, upper panel). Later in time, virus-like symptoms and GFP fluorescence were detected in the upper leaves of plants inoculated with PPV and PPV-VP3 chimera (Fig. 5B, lower panels), supporting the idea that IBDV VP3 is able to replace HCPro in a potyviral infection.

Western blot analysis of inoculated and systemically infected tissues confirmed the visual observations (Fig. 5C). Similar levels of accumulation of PPV-VP3 and wild type PPV were detected in the inoculated leaves. Viral CP was also detected in upper non-inoculated leaves of plants infected with PPV-VP3, albeit in somewhat lower levels than in wild type PPV-infected plants (Fig. 5C, lower panel). Further observations at later time of infection (38 dpi) showed that plant infected with PPV-VP3, but not those infected with wild type PPV, recovered from viral infection, displaying neither GFP fluorescence nor viral symptoms in new growing leaves (data not shown). The recovery process was confirmed by Western blot analysis of samples collected at 38 dpi, which showed an obvious drop in PPV CP accumulation in older leaves when compared with younger leaves in PPV-VP3-infected plants, a phenomenon that did not occur in plants infected with wild type PPV (Fig. 5D).

## Discussion

Viral RNAs, particularly in double-stranded conformation, are specifically recognized by a diverse battalion of sentinels that form part of the innate immune system of eukaryotes [10]. In many viral infections, dsRNA molecules are produced from their single-stranded counterparts as replication-intermediates or fold-back structures, or by action of RdRps, being then recognized by host factors for further activation of appropriate defence cascades. Interestingly, several viral families whose members have dsRNA genomes exist in the nature, which might demand additional protective measures to ensure efficient infection. The VP3 protein produced by members of the family *Birnaviridae*, is known to be involved in forming filamentous ribonucleoproteins that shield genomic dsRNAs [55]. In this report, we demonstrate that the VP3 proteins of several birnaviruses are also able to counteract RNA silencing by preventing formation of siRNAs from dsRNA silencing inducers.

Plants have been previously used to identify and study VSRs derived from vertebrate-infecting viruses given that the interpretation of results is not distorted by the presence of other dsRNA-related antiviral defences, like the interferon pathway [26], [27], [40], [69]. Thus, we made use of two co-agroinfiltration assays in *N. benthamiana* plants. In the first assay, RNA silencing is triggered by the sense transcript of the GFP reporter gene, which is converted into dsRNA by a host RdRp, while in the second, RNA silencing is induced by an inverted-repeated construct that directly forms a GFP-specific dsRNA. IBDV VP3 protected GFP mRNA from RNA silencing-mediated degradation in both systems (Fig. 1). We therefore conclude that VP3 acts downstream the dsRNA formation, which is in agreement with the dsRNA nature of birnaviral genomes.

Many VSRs bind dsRNA [33], [70], but not all dsRNA binding proteins have RNA silencing suppression activity [26], [29]. Whereas some VSRs bind long dsRNA molecules without size specificity and interfere with Dicer cleavage activity [70]–[74], others disturb silencing by specific sequestration of siRNAs [64], [75]–[78]. To establish the mechanism of action by which VP3 suppresses RNA silencing, we found that, in addition to its capacity to interact with long-dsRNAs [66], [67], IBDV VP3 can effectively bind ds-small RNA molecules of different sizes (Fig. 2B). This includes dsRNAs with a length of 21-nt, which is the typical siRNA size class produced from RNA viruses during the infection cycle. Moreover, whereas the plant VSR P1b, which suppresses silencing by specific interaction with 21-nt small RNAs [64], mainly affected the accumulation of secondary siRNAs, both primary and secondary GFP-derived siRNAs were barely detected in leaves expressing either IBDV VP3 or Influenza virus NS1 (Fig. 1B). These data support the idea that VP3 and NS1 could use a dual mode of action, which would be different to that used by P1b, based on perturbing Dicer-mediated cleavage of long dsRNAs to produce siRNAs whilst also blocking the incorporation of these siRNAs into RNA-induced silencing complexes. A similar mechanism of action has been suggested for the VSR B2 from the insect nodavirus *Flock house virus* [71], [79]. By using a mutagenic approach, we have identified a region of basic residues, called here as Patch 1, involved in the dsRNA binding activity of IBDV VP3 and shown that these residues are also relevant for RNA silencing suppression (Fig. 2C and Fig. 3). Whereas these results support the contribution of dsRNA binding to the RNA silencing suppression activity of VP3, they do not provide clues on the relative importance of long dsRNA protection and siRNA sequestration since all mutations had similar effects on the VP3 binding to the different tested RNA molecules (Fig. 2C and Fig. 3). Interestingly, whereas mutations in another region of basic residues, here called as Patch 2, produced a partial reduction in the capacity of IBDV VP3 to interact with both long and short dsRNAs, they did not affect the ability of this protein to suppress the degradation of GFP mRNA by silencing (Fig. 2C and Fig. 3). This result indicates that a diminished dsRNA binding is enough to counteract the silencing machinery, as previously observed for the VSR P1b [64], and supports the idea that the VP3 Patch 2 could also be involved in RNA interaction.

The expression of VSRs is the general strategy used by plant viruses to counteract antiviral RNA silencing and some of them, like the potyvirus PPV, are unable to establish an infection in the absence of an active VSR [80], [81]. Here, we found that IBDV VP3 is able to replace HCPro, the well-characterized VSR, in a PPV infection (Fig. 5). This result demonstrates the effectiveness of the RNA silencing suppression activity of IBDV VP3 to counteract host defences in a viral infection context. However, it is important to note that although the antisilencing activities of IBDV VP3 and PPV HCPro expressed from the PPV polyprotein are similar in an agroinfiltration assay (Fig. S2), the infection efficiency of PPV-VP3 chimera is somewhat lower than that of wild type PPV. *N. benthamiana* plants infected with this chimerical virus did eventually recover from the infection (Fig. 5D). These findings are in agreement with recent results obtained with PPV chimeras expressing different heterologous VSRs [82], supporting the idea that the capacity of a given VSR to replace HCPro in a potyviral infection does not rely only in the strength of suppressors, but also in the particular molecular mechanism that they use to suppress the RNA silencing.

It is well established that RNA silencing plays a key role in the antiviral innate immunity of invertebrate organisms [23], [24], [72], [83], and it has been recently demonstrated that antiviral silencing can terminate viral infection in adult insects [84]. Although a broad range of insect viruses are known targets of antiviral RNA silencing, VSRs have been identified only in positive sense strand RNA viruses [84], [85]. Interestingly, it has also been reported that RNA silencing is an efficient antiviral immune response against the birnavirus DXV [86], but the mechanism used by the virus to counteract this antiviral defence remained uncharacterized. Here, we provide the first evidence that the VP3 protein from DXV has RNA silencing suppression activity (Fig. 4), which suggests that it plays this counter defensive role during insect infections. The best-studied VSR of an insect virus, the B2 protein of alphanodaviruses [23], [71], [79], has a functional counterpart in fish betanodaviruses [87], [88]. Similarly, our data show that VP3 from IPNV, a fish infecting birnavirus, also has the ability to suppress the RNA silencing. It is worth to mention that even thought the primary amino acid sequence among these three VP3s is barely conserved, particularly remarkable in the case DXV VP3 [56], all of them interact with dsRNA without size specificity and counteract the RNA silencing (Fig. 4). Altogether, these results strongly supports the idea that silencing suppression is a conserved activity among different VP3 proteins and that RNA silencing could play a defensive role in viral infections of fishes and birds. In agreement with this postulate, an VSR from an avian herpersvirus has also been recently characterized [41].

Conclusive evidence demonstrating that an antiviral silencing mechanism operates in vertebrates is still lacking. Although early tests failed to find viral-derived siRNAs, the hallmark of antiviral silencing, in different infected organisms (reviewed by [89]), the availability of deep sequencing technologies has recently allowed not only the identification of siRNAs derived from a wide set of mammal-infecting RNA viruses, but also revealed their association with silencing effector complexes [13], [14]. These data, together with the observation that many mammal-infecting RNA viruses encode VSRs [42], suggest that RNA silencing of vertebrates is, to some extent, engaged in fighting against RNA viruses. It is important to note that the relationship between RNA silencing and viruses could be multifaceted in these organisms; hence, in addition to the possible cis-acting effect of viral-derived siRNAs, some of these molecules are able to target host genes involved in antiviral defense [90], [91]. Moreover, several host miRNA have been shown to contribute to virus resistance [34], [92]–[96], whereas others are essential for the replication of the virus [97]. In this intricate scenario, the contribution of VSRs is expected to be tightly regulated. In general, VSRs from mammal-infecting viruses also participate in the counter defense against other antiviral responses of the host, such as the interferon system, which add a further level of intricacy to the activity of these proteins [29], [31]. Cross-talk between RNA silencing and IFN-dependent defense pathways have been reported [13], [62], [94] and consequently the dsRNA-binding abilities of these VSRs could contribute to interference with both IFN- and RNA silencing-mediated antiviral defences in tandem [29]. Therefore, the dsRNA binding activity of IBDV VP3 could prevent genomic dsRNAs to induce RNA silencing, as well as modulate other dsRNA-induced antiviral defences. In agreement with this hypothesis, recent results indicate that IBDV VP3 inhibits PKR-dependent apoptosis induced by the expression of the viral protein VP2 (Busnadiego et al., *submitted*).

## Materials and Methods

### Plant Hosts

Agroinfiltration and viral infectivity assays were performed in *Nicotiana benthamiana* plants. All plants were grown in a greenhouse maintained at 16 hours light with supplementary illumination and a temperature range of 19–23°C.

### Plasmids

Recombinant baculoviruses (rBVs) expressing 6 × His-tagged versions of full-length IBDV VP3 as well as the VP3ΔC, which lacks the 36 C-terminal amino acid residues, were previously described [56], [66]. The construction of the rBV expressing a 6 × His-tagged version of VP3 Patch1 mutant (rBV hisVP3Patch1) was performed as follows. A 789 bp DNA fragment coding for a mutant version of VP3 with four aa substitutions (K99D, R102D, K105D and K106D, VP3MutPatch1) flanked by *BamH*I and *EcoR*I restriction sites, was generated by gene synthesis (Genscript) and inserted into the *EcoR*V site located in the multiple cloning site (MCS) of the pUC57 plasmid. This plasmid was digested with *BamH*I and *EcoR*I and the fragment containing the mutated VP3 ORF was purified and inserted into the MCS of the baculovirus transfer vector pFastBacHtb (Invitrogen), previously digested with the same restriction enzymes. The resulting plasmid (pFBhisVP3Patch1) was then used to generate the corresponding rBV making use of the Bac-to-Bac system, following the manufacturer’s instructions (Invitrogen). The generation of the rBV expressing a 6 × His-tagged version of VP3 Patch2 mutant (rBV hisVP3Patch2) that carries four point mutations (R159D, R168D, H198D and R200D) was done using the approach described above. The construction of the rBV expressing a 6 × His-tagged version of VP3 Patch1+2 mutant (rBV hisVP3Patch1+2) was performed by replacing the *BamH*I/*Xba*I fragment from the hisVP3Patch2 ORF by that of its hisVP3Patch1 counterpart. The construction of rBVs expressing the VP3 from DXV and IPNV was performed as follows. DNA fragments coding for the DXV VP3 and the IPNV VP flanked by *BamH*I and *EcoR*I were generated by gene synthesis (Genscript) and inserted into the *EcoR*V site of pUC57. Cloning of these ORFs into the baculovirus transfer vector pFastBacHtb to generate pFBhisVP3dxv and pFBhisVP3ipnv, and the generation of the corresponding rBVs, were carried out as described above.

To construct plasmids expressing the different versions of VP3 (wild type and mutants) and Influenza A NS1 in plants, GATEWAY technology (Invitrogen) was applied. Hence, pDONR-207 (Invitrogen) was used as donor vector, whereas pMDC32 (provided by Mark Curtis, University of Zurich) [98] and pNTAPi (provided by Michael Fromm, University of Nebraska) [99] were used as destination vectors. Primers and templates used for PCR amplifications to generate the different entry vectors are listed in Tables S1 and S2 in the supplemental material.

Expression vectors producing untagged CVYV P1b (pMDC32-P1b), untagged and TAP-tagged Influenza NS1 (pMDC32-NS1 and p35S-NTAP-NS1, respectively), untagged and TAP-tagged IBDV VP3 (pMDC32-VP3 and p35S-NTAP-VP3, respectively), TAP-tagged mutant versions of IBDV VP3 (p35S-NTAP-VP3patch1, p35S-NTAP-VP3patch2, p35S-NTAP-VP3patch1+2 and p35S-NTAP-VP3ΔC), TAP-tagged IPNV VP3 (p35S-NTAP-VP3ipnv) and TAP-tagged DXV VP3 (p35S-NTAP-VP3dxv) were constructed by LR clonase reactions between the corresponding pDONR entry vectors and either the destination vectors pMDC32 or pNTAPi (Table S1). Plasmid pDONR-P1b and p35S-NTAP-P1b were previously described [63].

Cloning of the IBDV VP3 ORF in a PPV full-length cDNA yielding pICPPV-VP3 made use of an intermediate clone, pGEMT-p1p3, and the full-length cDNA clone pICPPV-NK-GFPn, according to the procedure described by Maliogka et al. (manuscript submitted for publication). Primers and templates used for IBDV VP3 amplification are listed in Tables S1 and S2. pICPPV-NK-GFPn (P. Sáenz, M.R. Fernández-Fernández and J.A. García, unpublished results), a PPV full-length cDNA clone derived from pICPPV-NK-GFP (Fernández-Fernández, 2001 #3289}, and pICPPV-ΔHC [80] were used as control. The accuracy of all the constructs was verified by restriction digestion analysis, as well as DNA sequencing of all regions derived from PCR amplifications.

*A. tumefaciens* C58C1 strains carrying p35S:GFP [100] plus pCH32 [101], and p35S:GF-IR [102] were kindly provided by David Baulcombe (University of Cambridge, United Kingdom).

### Agroinfiltration and Fluorescence Imaging

*N. benthamiana* plants were infiltrated with *A. tumefaciens* C58C1 strain carrying the indicated plasmids as previously described [103]. GFP fluorescence was observed under a Leica MZ FLIII fluorescence microscope with excitation and barrier filters of 480/40 and 510 nm, respectively. Pictures of GFP were caught with an Olympus DP 70 camera and the software DP Controller and DP manager.

### RNA Extraction and Northern Blot Analysis

Samples of large and small RNAs were prepared from agroinfiltrated leaf patches and subjected to Northern blot analysis as previously described [103]. GFP siRNA were detected with ^32^P-labeled GF and P riboprobes, which were prepared by transcription with SP6 RNA polymerase from *SacII*-linearized pGEMT-GF and pGEMT-P, respectively. These plasmids contain the nt 4 to 403 (GF) and 404 to 717 (P) of the GFP gene cloned in pGEM-T.

### Expression and Purification of Different VP3 Proteins from Insect Cells

Procedures to express recombinant 6xHis-tagged VP3s in insect cells by using rBVs as well as to purify the polypeptides by metal-affinity chromatography were previously described [52].

### Gel Filtration Analysis

Affinity-purified 6xHis-tagged VP3s were analyzed by gel filtration using a fast protein liquid chromatography system (ÄKTA FPLC, Pharmacia) with a Superdex-200 5/150 column (Pharmacia) previously calibrated with catalase (158 kDa), serum albumin (68 kDa), ovoalbumine (50 kDa) and chymotrypsinogen A (21 kDa). Column equilibration and chromatography were performed at a flow rate of 0.5 ml/min in a buffer consisting of 20 mM Tris-HCl, pH 7.5, 150 mM NaCl at 4°C.

### Electrophoretic Mobility Shift Assay (EMSA)

Synthetic ds-siRNA with 2-deoxinucleotides 3′ overhands (5′CUUACGCGAGUCUUCGATT3′/5′UCGAAGUACUCAGCGUAAGTT3′) (Sigma) were labelled with γ-^32^P-ATP using T4 PNK (Promega). IBDV genomic double-stranded RNAs were prepared accordingly to Luque et al [55].

Either labelled siRNA (2 nM final concentration) or genomic IBDV RNAs (7.5 nM final concentration) were incubated with different amounts of the indicated affinity chromatography-purified proteins for 30 min at room temperature in a reaction mixture (20 µl) containing small RNA-binding buffer (10 mM TrisHCl pH8, 10 mM Glycine, 2 mM DTT) or long dsRNA-binding buffer (50 mM TrisHCl pH8, 150 mM NaCl, 0.5µg/ul BSA), respectively. After incubation, protein-small RNA and protein-long dsRNA complexes were resolved on 5% polyacrylamide-containing 0.5×Tris-borate-EDTA gels and 1% agarose-containing 0.5×Tris-borate-EDTA gels, respectively. Polyacrylamide gels were dried and exposed to X-ray sensitive films. Agarose gels were stained with ethidium bromide and exposed under UV light.

### Biolistic Inoculation

The Helios Gene Gun System (Bio-Rad, Hercules, CA, U.S.A.) was used for biolistic inoculation. Microcarrier cartridges were prepared from 2 different clones per construct, with 1.0 µm gold particles coated with the different plasmids at a DNA loading ratio of 2 µg/mg of gold and a microcarrier loading of 0.5 mg/shooting. Helium pressure of 7 bars was used for shooting plants. Each cartridge was shot twice onto two leaves of each plant.

### Western Blot of Infected Plants

Tissue samples of inoculated leaves were harvested under UV light from GFP expressing foci, whereas tissue of upper non-inoculated leaves were harvested from the indicated whole leaves. Control samples corresponding to non-infected leaves were taken from equivalent areas. Preparation of protein samples, SDS-PAGE electrophoresis, and electroblotting were done as previously described [103]. Specific proteins were detected using anti-IBDV VP3 rabbit serum [104] or anti-PPV CP rabbit serum, as primary antibodies, and horseradish peroxidase (HRP)-conjugated goat anti-rabbit IgG (Jackson) as secondary reagent. The immunostained proteins were visualized by enhanced chemiluminiscence detection with a LifeABlot kit (Euroclone). Ponceau red staining was used to check the global protein content of the samples.

## Supporting Information

Figure S1 Amino acid changes in Patch 1 and Patch 2 domains do not alter the proper dimerization of IBDV VP3. Analytical gel-filtration assays of wild type IBDV VP3 and its Patch 1 and Patch 2 mutant derivatives. The arrow indicates the elution position of IBDV VP3 dimer.
(TIF)

Figure S2 Efficient anti-silencing activities of IBDV VP3 forming part of the PPV polyprotein. (A) Schematic representation of the GFP and viral-derived constructs used in the RNA silencing suppression test in *N. benthamiana* plants. Black arrows indicate self-cleavages by the corresponding viral proteases, whereas the grey arrow indicates a cleavage *in trans* by the action of NIaPro. GFP fluorescence photos taken under an epifluorescence microscope (B) and Northern blot analyses of GFP mRNA (C) from leaves of two independent plants collected at 6 days post agroinfiltration (two different clones of the indicated VSR were separately analyzed). EtBr-stained rRNA is shown as loading control.
(TIF)

Table S1 List of primers and templates used for PCR in the construction of different entry vectors.
(DOC)

Table S2 Sequence of PCR primers used in the plasmid constructions.
(DOC)
