# Supplementary figures and images for: The VP3 Factor from Viruses of Birnaviridae Family Suppresses RNA Silencing by Binding Both Long and Small RNA Duplexes
Source: PLoS One. 2012 Sep 25;7(9):e45957. doi: 10.1371/journal.pone.0045957 (PMC3458112; doi:10.1371/journal.pone.0045957)

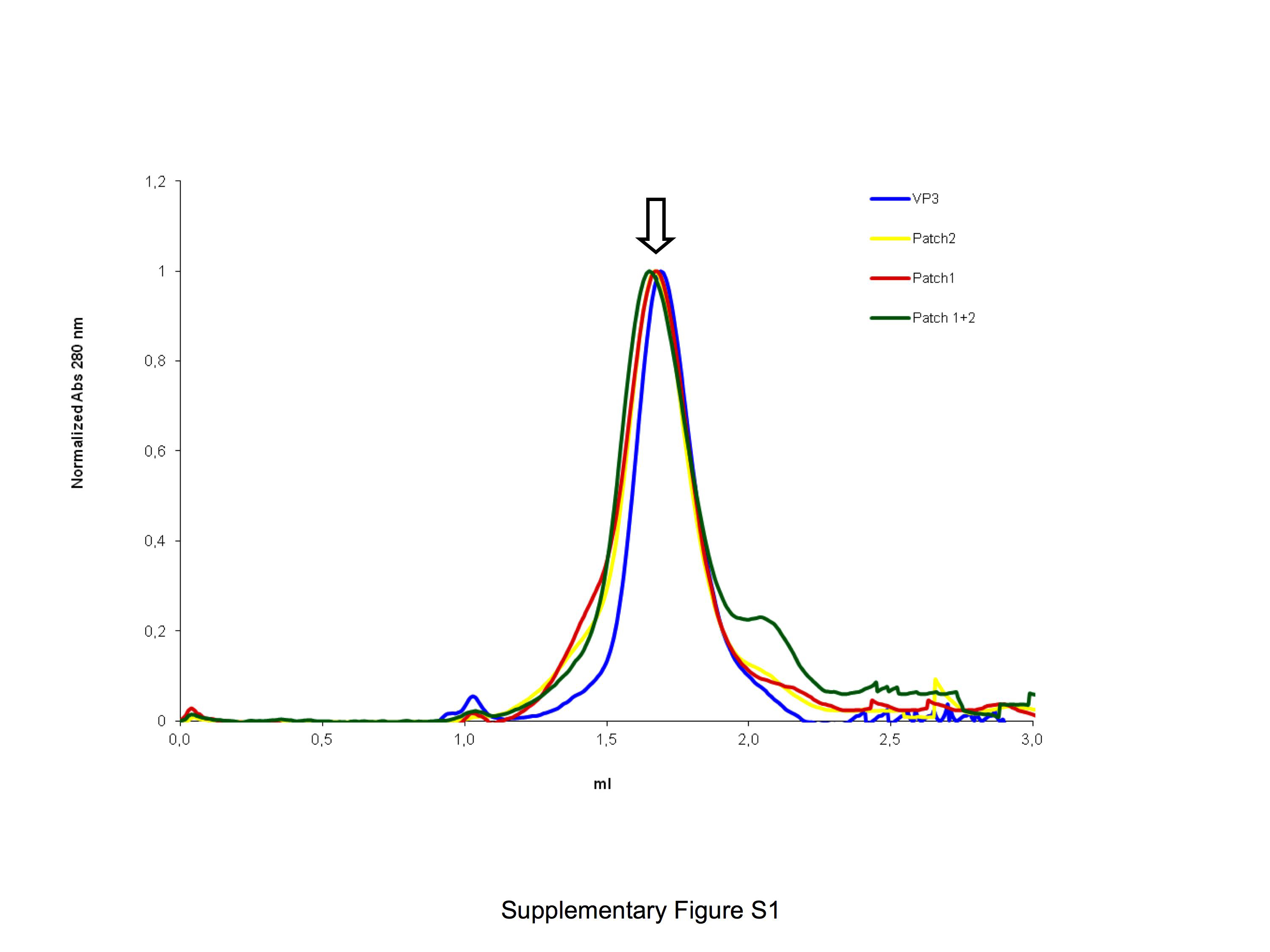

Supplement: Figure S1 — Amino acid changes in Patch 1 and Patch 2 domains do not alter the proper dimerization of IBDV VP3. Analytical gel-filtration assays of wild type IBDV VP3 and its Patch 1 and Patch 2 mutant derivatives. The arrow indicates the elution position of IBDV VP3 dimer. (TIF) [file pone.0045957.s001.tif]

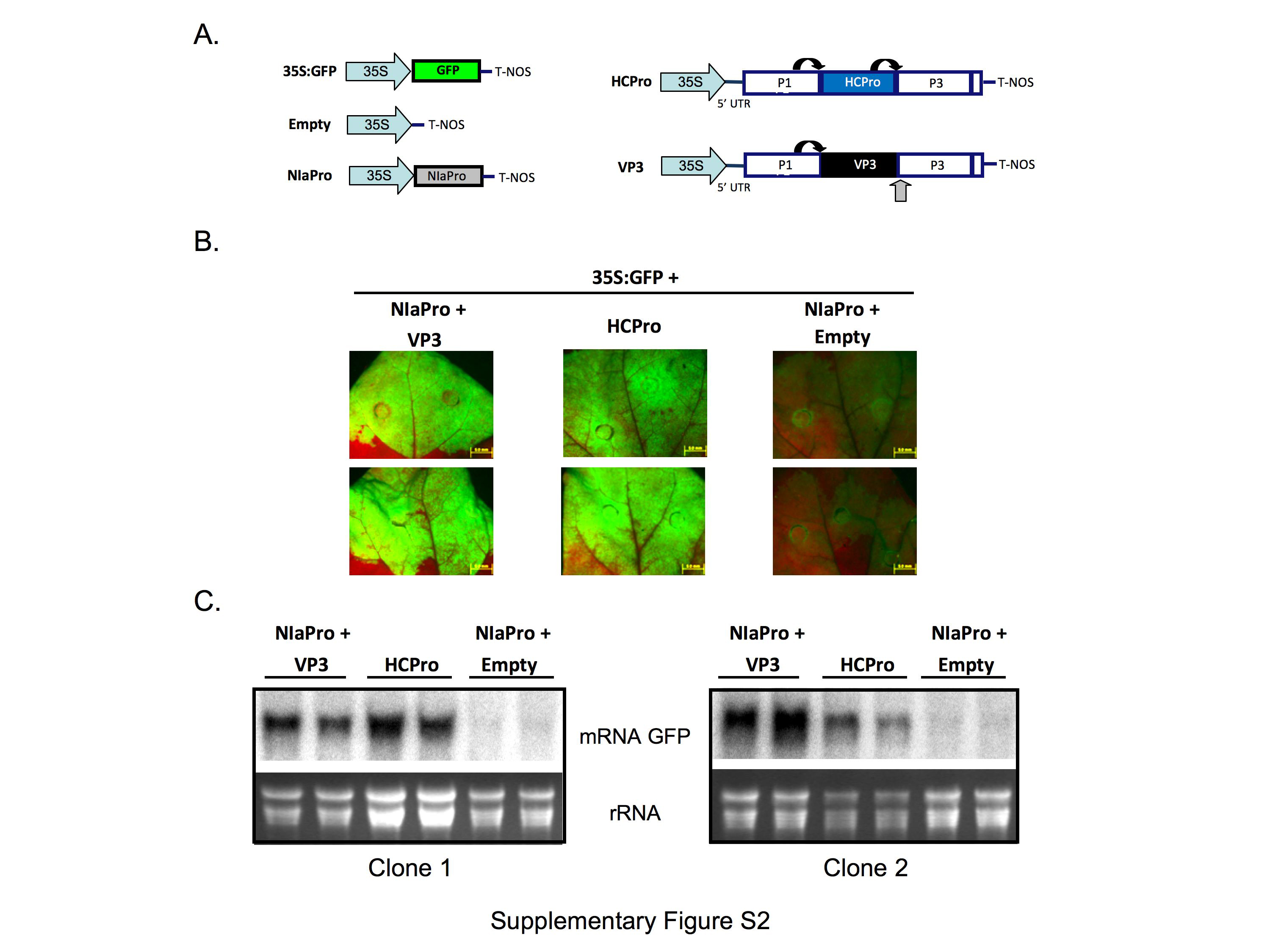

Supplement: Figure S2 — Efficient anti-silencing activities of IBDV VP3 forming part of the PPV polyprotein. (A) Schematic representation of the GFP and viral-derived constructs used in the RNA silencing suppression test in N. benthamiana plants. Black arrows indicate self-cleavages by the corresponding viral proteases, whereas the grey arrow indicates a cleavage in trans by the action of NIaPro. GFP fluorescence photos taken under an epifluorescence microscope (B) and Northern blot analyses of GFP mRNA (C) from leaves of two independent plants collected at 6 days post agroinfiltration (two different clones of the indicated VSR were separately analyzed). EtBr-stained rRNA is shown as loading control. (TIF) [file pone.0045957.s002.tif]
