# Supplementary material for: The VP3 Factor from Viruses of Birnaviridae Family Suppresses RNA Silencing by Binding Both Long and Small RNA Duplexes
Source: PLoS One. 2012 Sep 25;7(9):e45957. doi: 10.1371/journal.pone.0045957 (PMC3458112; doi:10.1371/journal.pone.0045957)
Supplement: Table S1 — List of primers and templates used for PCR in the construction of different entry vectors. (DOC) [file pone.0045957.s003.doc]

**Table S1.** List of primers and templates used for PCR in the construction of different entry vectors.

| **Entry plasmid** | **Forward** ^a^ | **Reverse** ^a^ | **Template** |
| --- | --- | --- | --- |
| **pDONR-NS1** | # 1303 | # 1304 | 35S-NS1 (1) |
| **pDONR-VP3** | # 1305 | # 1306 | pVOTE.1/VP3 (2) |
| **pDONR-VP3patch1** | # 1305 | # 1306 | pFBhisVP3Patch1 |
| **pDONR-VP3patch2** | # 1305 | # 1306 | pFBhisVP3Patch2 |
| **pDONR-VP3patch1+2** | # 1305 | # 1306 | pFBhisVP3Patch1+2 |
| **pDONR-VP3ΔC** | # 1305 | # 1562 | pVOTE.1/VP3 (2) |
| **pDONR-VP3ipnv** | # 1633 | # 1634 | pFBhisVP3ipnv |
| **pDONR-VP3dxv** | # 1635 | # 1636 | pFBhisVP3dxv |

^a^ The sequences of the primers are shown in the Table S2.

1. **Delgadillo, M. O., P. Sáenz, B. Salvador, J. A. García, and C. Simón-Mateo.** 2004. Human influenza virus NS1 protein enhances viral pathogenicity and acts as an RNA silencing suppressor in plants. J. Gen. Virol. **85:**993-999.

2. **Fernández-Arias, A., S. Martínez, and J. F. Rodríguez.** 1997. The major antigenic protein of infectious bursal disease virus, VP2, is an apoptotic inducer. J. Virol. **71:**8014-8018.
