# Supplementary material for: The VP3 Factor from Viruses of Birnaviridae Family Suppresses RNA Silencing by Binding Both Long and Small RNA Duplexes
Source: PLoS One. 2012 Sep 25;7(9):e45957. doi: 10.1371/journal.pone.0045957 (PMC3458112; doi:10.1371/journal.pone.0045957)
Supplement: Table S2 — Sequence of PCR primers used in the plasmid constructions. (DOC) [file pone.0045957.s004.doc]

**Table S2.** Sequence of PCR primers used in the plasmid constructions.

| Primer | Sequence (5’-3’) ^a^ |
| --- | --- |
| # 1303 | ggggacaagtttgtacaaaaaagcaggctccATGGATTCCAACACTGTGTCAAG |
| # 1304 | ggggaccactttgtacaagaaagctgggTTAATCAGCCATCTTATCTCTTCGAAC |
| # 1305 | ggggacaagtttgtacaaaaaagcaggctccATGGCATCAGAGTTCAAAGAGACCC |
| # 1306 | ggggaccactttgtacaagaaagctgggTTACTCAAGGTCCTCATCAGAGACGG |
| # 1562 | ggggaccactttgtacaagaaagctgggTTAGCGATGCTTCATCTCCATCG |
| # 1633 | ggggacaagtttgtacaaaaaagcaggctccATGGCTTCCGGTATGGACGAGGAACTGC |
| # 1634 | ggggaccactttgtacaagaaagctgggTTAGACTTCGGCGTTGTCGCCAGAAGG |
| # 1635 | ggggacaagtttgtacaaaaaagcaggctccATGGCTAAGCCTATCTCCGAGGAAACC |
| # 1636 | ggggaccactttgtacaagaaagctgggTTAGACGATGTCGTTGTCCTCACCACG |

^a^ GATEWAY recombination sequences are indicated in lower case.
